# Promoting Health Behavior Change in the Preconception Period: Combined Approach to Intervention Planning

**DOI:** 10.2196/35108

**Published:** 2022-04-28

**Authors:** Jodie Scott, Melissa Oxlad, Jodie Dodd, Claudia Szabo, Andrea Deussen, Deborah Turnbull

**Affiliations:** 1 School of Psychology The University of Adelaide Adelaide Australia; 2 Robinson Research Institute Adelaide Medical School The University of Adelaide Adelaide Australia; 3 Department of Perinatal Medicine Women’s and Babies Division Women’s and Children’s Hospital Adelaide Australia; 4 School of Computer Science The University of Adelaide Adelaide Australia

**Keywords:** intervention mapping, preconception, behavior change, healthy lifestyle, maternal health, weight management

## Abstract

**Background:**

Half of women begin pregnancy above the healthy weight range, increasing the risk of complications and adversely affecting the lifelong health of their babies. Maternal obesity remains the strongest risk factor for offspring obesity across childhood, adolescence, and adulthood. Previous research suggests that women should be encouraged to be within a healthy weight range before conception to improve health outcomes.

**Objective:**

We outlined the intervention planning and design process to develop an evidence-informed eHealth intervention to promote weight management. The intervention, based on psychological theories and behavior change techniques, has been developed for women affected by overweight or obesity who intend to become pregnant. The *Begin Better* web application is part of an integrated program being evaluated in a clinical trial to assess if weight management *before* pregnancy can influence clinical outcomes for mothers and babies.

**Methods:**

Our intervention development process was guided by intervention mapping and person-based methods. This study documents steps 2 to 4 of a 6-step iterative intervention mapping approach informed by the Information-Motivation-Behavioral Skills model and the findings of a previous interview study. We defined behavior change objectives for each of the Information-Motivation-Behavioral Skills behavioral determinants as well as theory-based behavior change techniques and practical strategies. We also used persuasive system design principles to assist in translating these strategies into a digital environment.

**Results:**

The resultant intervention comprises nutritional and physical activity content along with psychological strategies, which are notably absent from mainstream weight management programs. Strategies to increase motivation, garner social support, and promote self-care are integral to maintaining engagement with the intervention, which aims to improve lifestyle behaviors and enhance well-being. Important elements include tracking mechanisms for percentage progress toward goals to enable feedback on behaviors and outcomes; in-application messages of praise on entry of goals or habits; and strategies to prompt habit formation and action planning via small, easily achievable steps toward positive change.

**Conclusions:**

Design decisions and processes for idea generation about intervention content, format, and delivery are often not reported. In this study, we respond to this gap in the literature and outline a process that is potentially transferable to the development of other interventions.

## Introduction

### Background

Women who begin pregnancy above the healthy weight range (BMI ≥25.0 kg/m^2^) can experience a range of complications such as gestational diabetes mellitus, pre-eclampsia, premature birth, and stillbirth and have a higher risk of fetal malformations [1]. In addition, babies born to women above the healthy weight range are more likely to be born with high birth weight and experience a range of health conditions—including obesity—across childhood and into adulthood [2]. The resulting costs to the health system and society are considerable [3]. Previous research suggests that being within the healthy weight range should be encouraged before pregnancy to improve outcomes [1]. Although clinical weight management interventions can be rigorous, they are often time- and resource-intensive. Interactive communication technology in the form of an eHealth application offers a low-cost, high-reach, and potentially scalable solution. Such technologies also seek to engage the intended population group—women of childbearing age—with a product that is enjoyable to use and effective in promoting behavior change.

This paper outlines the intervention planning approach used to develop the *Begin Better* eHealth intervention. The web application is part of a multicomponent intervention that is being evaluated via a clinical trial to establish whether weight management *before* pregnancy can influence clinical outcomes for women and their babies. Although theories often describe what is required to prompt healthy behaviors, they rarely describe *how* intervention techniques induce these changes [4,5]. In addition, design decisions and idea generation about intervention content, format, and delivery are often not discussed within frameworks or reported widely [6]. Researchers also acknowledge that scientific knowledge is too often valued over the practical and social wisdom of experience-based contextual knowledge [6]. Furthermore, it is thought that a person-based approach is crucial in determining which intervention design features will be most acceptable and effective for a particular population and context [7,8].

Intervention mapping (IM) is a method used to devise health promotion programs that enable effective decision-making at each development step [9]. The planning process integrates theory and empirical findings with the collection and use of new data to develop interventions [9]. IM has been used successfully across programs for diabetes prevention and weight control, chronic disease self-management, and complex behavioral interventions for diet and physical activity [10].

It is widely recognized that selecting appropriate behavior change techniques (BCTs) can greatly enhance the effectiveness of interventions [11]. A recent meta-analysis of 46 postpartum weight management interventions found that the most successful behavioral strategies for decreased energy intake were *problem-solving*, *goal-setting*, *reviewing goals*, *feedback on behavior*, *self-monitoring of behavior*, *behavioral substitution,* and *credible source* [12]—all predominantly related to self-regulation. Some evidence exists that including a greater number of BCTs predicts greater behavior change in some contexts [13], whereas this association was not found elsewhere [12]. Meta-analyses have found greater effects when *self-monitoring* was combined with other self-regulation techniques such as explicit *goal-setting*, *feedback on goal progress*, and *action planning* [14-16]. A weight loss randomized controlled trial (RCT) found that *action planning* had the highest “usefulness” rating of all self-regulation intervention components, with daily action plans noted as being particularly effective [15]. However, much of the existing research is not specific to preconception women. Several studies related to maintenance of behavior change [17,18], including our previous work [19], have also emphasized the need for ongoing positive motivational support and encouragement—a common barrier to long-term adherence.

Interventions that provide a person-centered and autonomy-supportive communication style such as those based on self-determination theory and motivational interviewing are associated with long-term efficacy [20]. Despite this, motivational interviewing remains challenging to implement in a web-based environment [21]. Although behavior change interventions with a theoretical background are more effective than those that are not theory-informed, translation difficulties mean that theory does not underpin many developed interventions [22]. In addition, theory-based interventions are often poorly reported, making replication or adaptation difficult. It is often unclear how the BCTs have been implemented or which ones are most effective in promoting positive change [5] as techniques are rarely implemented in isolation.

Researchers have suggested that one of the most important ways to improve health care is through the use of persuasion [23]. Persuasive design uses interactive technology to change users’ attitudes, behavior, or both [24]. A systematic review of 81 web-based interventions found that higher levels of interaction significantly predicted greater adherence [23]. In addition, a large-scale study of 568 participants investigated the perceived effectiveness of various individual strategies in motivating behavior change [25]. *Suggestion* was perceived as the most persuasive as it increases confidence for change, followed by *praise, self-monitoring*, and *reminder* [25].

### Our Research Objective

Our primary objective is to plan, design, and develop a behavior change web application as part of a multicomponent intervention—the *Begin Better* trial for preconception weight management. Although the application also includes general preconception care information, nutrition and physical activity sections, and a recipe database, this study is primarily concerned with the *Mind* modules of the application. We outline the application of methods adapted from IM [9] and person-based approaches [7] to develop an evidence-informed eHealth intervention to promote weight management. The intervention, based on psychological theories and BCTs, has been developed for women affected by overweight or obesity who intend to become pregnant. Begin Better is part of an integrated program being evaluated in a clinical trial to assess whether weight management *before* pregnancy can influence clinical outcomes for mothers and babies. Participants will, at least initially, also have access to face-to-face sessions with a health coach to complement the strategies used in the application and build on the personalized and collaborative approach to aid motivation.

Although intervention effects can often diminish over time or when support ends, the intent of the Begin Better program is a lifestyle change that women can sustain in the long term. Contrary to the *initiation* of change, theories of behavior change and weight loss *maintenance* focus on strategies such as sustained motivation, self-regulation, psychological and physical resources, habit formation, and environmental and social influences [17,26]. These reflect how health changes can be maintained over time and in different contexts. At least one sustained motivator, which may include enjoyment, satisfaction with outcomes, self-determination, or a sense of alignment with values or beliefs, is required to maintain the new behavior [17]. This aspect is especially salient as our previous interview study found that all participants had made previous attempts to lose weight but could not maintain the changes in the long term [19]. We intend to build motivation, skills, and self-efficacy so women can make sustainable lifestyle changes.

## Methods

### Overview

We aim to systematically describe the adapted IM and person-based process and the evidence-informed and practical strategies used to develop the *Begin Better* intervention. Begin Better targets cognitive restructuring around weight management, stress reduction, physical activity, and healthy eating behaviors. A person-based approach was used in initial interviews with 23 target women [19] to ground the design in an in-depth understanding of users’ beliefs and psychosocial contexts. This process is particularly relevant in increasing engagement with digital interventions [7] and complements the theory- and evidence-based approaches to intervention development.

### IM Approach

We used the 6-step IM protocol for behavior change interventions as a basis for our intervention planning [9]. Although it is an iterative process, this study reports on steps 2 to 4. Step 1 (needs assessment) occurred via the previous qualitative interview study with 23 women aged 23 to 48 years who were above the healthy weight range and wanted to make lifestyle changes [19]. Exploring the women’s emotional and social contexts, knowledge, motivations, skills, and self-efficacy in changing health behavior, this qualitative study identified the personal determinants of change that drove the intervention development and enabled the production of a logic model.

Step 2 involved defining the intervention objectives for each of the behavioral determinants. Change objectives were drafted by the first author (JS) informed by the barriers, facilitators, and needs identified in the interview study, and a consensus was reached with other authors (DT and MO). In a person-based approach, these reflect the psychosocial characteristics and context of the intended users.

In step 3, BCTs and practical strategies were identified to address the objectives based on appropriate theory and evidence. The selection of BCTs was based on what has been deemed effective within each construct domain [27] and what is known to be effective in previous studies in similar contexts [12]. Change methods were operationalized to meet the parameters for effectiveness set out in the IM behavior change tables [9], where crossover occurs with BCTs [28] used in the intervention. Examples include *goal-setting*, whereby the women are prompted to set challenging but achievable goals. Similarly, *providing cues* enables the women to have the autonomy to select their own cues for healthier habits. Brainstorming and lateral thinking were used to consider practical strategies that were engaging and met the objectives of the intervention while still addressing the parameters. Where parameters were undefined, we used discretion to apply assumptions for effectiveness given the situation and context. Persuasive principles were also chosen to assist in translating design features for greater user engagement, enjoyment, and effectiveness [29].

Step 4 comprised refining the program structure and producing content. A multidisciplinary team comprising academic researchers, clinical and health psychologists, dietitians, designers, computer scientists, web developers, and women in the target population were consulted to develop the intervention. The first author (JS) completed the planned content outlines for the *Mind* modules and developed draft content along with annotated mock-ups of interactive components. A stepped approach of psychological skill building was adopted throughout the 5 modules, with plans integrated for effective delivery within the constraints of the medium.

Mind module content and interactive components were reviewed by an obstetrician (JD), clinical and health psychologists (DT and MO), a midwife, a dietitian, a nutritionist and health behavior researcher, academic researchers (AD), and clinical psychologists not associated with the project to ensure clinical accuracy and comprehension. Feedback was provided regarding simplifying some psychological concepts for our target audience and further use of metaphors to explain difficult or unfamiliar ideas. Minor grammatical edits were also made. The web programmer reviewed the overall structure and interactive elements to ensure that they were feasible to build into the application within the constraints of the medium. Drafts were reviewed and discussed with all authors, and agreement was reached on final content.

Videos and podcasts were recorded and edited by members of the research team with skills in this area. A PhD-qualified registered nutritionist and health behavior researcher—who is not an author of this paper—presented all videos and podcasts to eliminate bias in the evaluation process. The web programmer integrated the interactive tasks at key points within each video aligned with the appropriate session of the module.

Informal pilot testing of various components provided direction for the final interface. A total of 9 women in the target age group provided feedback on branding and design options for the look and feel of the application. General feedback was that the fun and relaxed designs depicting warmth and care were favored over the more clinical representations of health—in part because of the highly stigmatizing nature of being overweight in clinical settings.

Various sources of knowledge are often valued differently and can change across the development process [6]. Design decisions were driven primarily by the new interview data from the target group, with theory and empirical evidence, and informal pilot-testing providing secondary sources of guidance. Although all sources of knowledge were valuable, the stakeholder views allowed us to tailor the content to the personal determinants of our target population and to suit the context of their lives. These were women in a busy phase of life, time-poor, and often with competing work and child-rearing demands. Some belonged to lower socioeconomic groups, with limited access to information and resources. Many had also tried unsuccessfully to lose weight previously, so we knew that they required a less prescriptive approach to weight management with flexible options that could be maintained in the longer term. Some of the women struggled with comorbidities that could be improved with lifestyle changes. Many in this life phase also told us that their partners or children created barriers to healthier habits, so strategies that included partners for support were prioritized given that the intent was to conceive a child.

The intervention content and structure were also informed by published theories and evidence to enhance effectiveness. Learnings from previous complex intervention development projects also guided decision-making [6,30,31]. In addition, consideration was given to developing an eHealth solution that would be feasible to develop and deliver within time, resource, and funding constraints.

Finally, steps 5 and 6 involve planning the implementation, delivery, and evaluation. Although these stages are not reported in this paper, considerations for implementation begin with the initial needs assessment and continue throughout all steps. Change methods and practical strategies were carefully selected to support implementation. Forward planning included ensuring that all aspects of delivery satisfied the change objectives and implementation outcomes and could be easily tracked in evaluation studies. Planning also established that the intervention could be readily adapted following feedback or scaled up for future use if proven effective through RCT.

The iterative process means that steps are often not cycled through in a systematic linear manner; instead, it was necessary to readdress aspects of previous steps and refine the program to ensure that intervention strategies were feasible. An example is that we scaled back personalization aspects as developing a complex level of tailored content was not achievable within the resources available—therefore, BCT methods, persuasive system design (PSD), and practical strategies were revised accordingly.

### Information-Motivation-Behavioral Skills Model

We used the Information-Motivation-Behavioral Skills model [32] for the interview study that informed the planning process. This allowed us to address any knowledge, motivation, and skill deficits of the target population. The model’s simple structure allows for the easy translation of constructs into intervention components. It provides a framework for defining intervention objectives and candidate change techniques that may be key to the effectiveness of the final program [11]. The model has been well-tested in changing health behaviors and is highly applicable to weight management [33].

### BCTs Used

The BCTs we used in the intervention were classified and described using the Behavior Change Technique Taxonomy [28], consisting of 93 techniques clustered across 16 categories (Multimedia Appendix 1 [28]).

### PSD Model Principles

We used the PSD framework [34] to develop the eHealth intervention’s technology elements, features, and interactivity to increase participant engagement and adherence. The framework proposes persuasive principles across 4 categories: primary task support (supporting users to perform the intended tasks), dialogue support (providing feedback that directs users toward healthier behaviors), system credibility support (supporting the development of trustworthy systems), and social support (motivating users through social influence; Multimedia Appendix 2 [34]). We chose this framework for the design principles’ particular relevance to well-being, nutrition, and physical activity given their success in these areas [29].

### Ethics Approval

The interview study that informed the planning process was approved by the Women’s and Children's Health Network (HREC/19/WCHN/108) and the University of Adelaide Human Research Ethics Committee (33863).

## Results

### Overview

**Figure 1:**
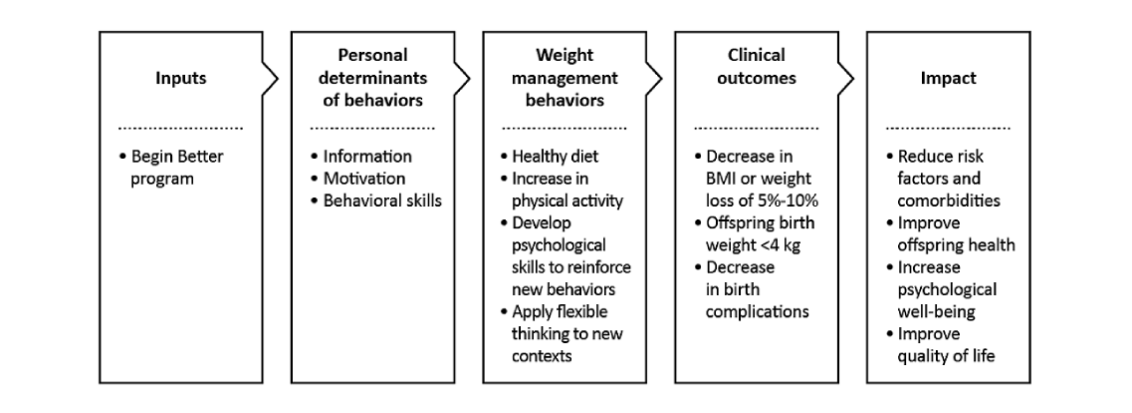
Logic model of the Begin Better intervention.

Compared with the reductionist philosophy often used in weight management programs, the Begin Better intervention uses a holistic approach informed by the biopsychosocial model [35], which considers the biological, psychological, and socioenvironmental factors integral to health behavior change. A logic model of the intervention based on the personal determinants of the Information-Motivation-Behavioral Skills model; the desired weight management behaviors; clinical outcomes for the program; and the health, social, and emotional impact is shown in Figure 1. Multimedia Appendix 3 describes the change objectives, BCTs, PSD principles, and practical strategies used throughout the intervention aligned with each of the determinants.

Some of the more important techniques supported by strong evidence of effectiveness and with relevance to our context are described below, with the BCTs and PSD principles highlighted in italics.

### BCTs Used

BCTs are used throughout the eHealth intervention. Techniques known to be the most effective, such as *goal-setting* and *self-monitoring*, are used early in the intervention. Women are guided to set goals that align with their values and encouraged to set a weight goal and several whole-person goals related to health, social, or mental well-being. Self-monitoring of weight and whole-person goals occurs within the application, with the ability to add, review, or update goals and habits as they are achieved or maintained to build self-efficacy for change. Bar charts that track percentage progress toward goals and a graph showing the overall trend line enable timely *feedback on behavior* and *outcomes* to increase motivation. Users are also encouraged to consider health benefits and measures of success beyond just the number on the scale.

One of the more effective BCTs is providing *information about health consequences*. Interviews with target women demonstrated that knowledge of the impacts of their weight status on themselves and their babies was alarmingly low. By highlighting the benefits of a healthy lifestyle before pregnancy in a nonjudgmental and supportive tone, we hope to provide women with added motivation. The language used was conversational rather than clinical, using terms such as *benefits of weight management* rather than noting the *risks*. Similarly, women are encouraged to consider the benefits of a healthy lifestyle beyond just weight management, with *information about emotional consequences* and *information about social and environmental consequences* provided. This was done via an animated sequence that highlighted the benefits not just for their babies but also for themselves—including psychosocial benefits such as more energy, positive mood changes, and being a role model for others.

The intervention also includes aspects of *nudge* theory, whereby small, simple *micronudges* are used to initiate healthy change rather than complex or restrictive regimes. For example, *restructuring the physical environment* prompts small changes in the women’s choice architecture (eg, placing healthy food within easy eyeshot and reach, keeping sneakers by the front door as a reminder to take a walk, or preloading a meditation app that is visible each time the phone is used). These strategies make healthier choices easier to make.

Research also indicates that habits are important drivers of behavior [36], especially healthy eating and physical activity. *Habit formation* is promoted through ideas and inspiration to implement new healthy habits into women’s daily routines. These techniques can influence behavioral flexibility and have been effective in clinical trials [37,38]. As noted previously, effective interventions need to focus on prompting action rather than merely providing motivation [4,5]. *Action planning* is an important aspect of the intervention; women will create and upload habit action plans to the application.

*Social support* and encouragement remain important motivators as many women in our previous interview study reported that interpersonal challenges had affected their motivation toward lifestyle change [19]. An opt-in *buddy system* is offered, whereby participants are paired with another woman in the program to reciprocate motivation and encouragement. Some women in the interview study noted the motivation and satisfaction they would garner from helping a peer along their journey to health [19]. Buddies may also function as accountability partners who check in and keep each other accountable to their goals and problem solve issues together. Women’s attention is also drawn to *identification of self as role model* for those around them. Strategies are promoted to encourage healthy change across their entire family.

Other BCTs important for mental well-being and self-efficacy, such as *problem-solving* and relapse prevention, are also used as strategies to maintain motivation and increase psychological flexibility. *Repetition and substitution*, including *habit formation,* are considered integral to behavior change. Women are encouraged to integrate microhabits into their daily routine—small changes they can repeatedly implement until they become automatic. Substitution ideas are also offered for less healthy foods and behaviors.

### PSD Model Principles

Our intervention incorporates the following PSD techniques. *Tunneling* guides users through the process of behavior change without overwhelming them. Key *Mind* modules, unlocked at fortnightly intervals, include various psychological techniques to create a healthier lifestyle. These are aligned with key *Nutrition* modules to deliver a cohesive program with a natural progression of skill building. Some nutrition and physical activity content is also free-roam so that the application provides information and engaging content at all times. Topics include how to read food labels, portion control, practical tips for simple meals, and reducing sedentary time.

In-application alerts are provided to motivate and draw attention to modules as they are unlocked. For example, if women do not upload their weight for a week, *reminders* prompt a weigh-in for self-monitoring. *Praise* is offered when goals are uploaded and action plans are set with in-application messages such as “Great work with the values. Let’s discuss them at our next catch-up” and “It feels great to have plans in place! Now for the benefits to flow!” Women are also prompted to reflect on their progress.

*Reduction* is a key technique used to simplify complex behaviors into manageable tasks that women can easily integrate into their lives. Women in our interview study cited complexity and lack of flexibility as reasons why previous weight management programs were unsustainable [19]. *Suggestion* is also used, with ideas and inspiration for small daily changes that can be made to promote healthier behaviors. A database of easy, fast, dietitian-approved recipes is also included to reduce the cognitive load associated with making daily food decisions.

Dialogue support strategies such as *Suggestion* are integrated, providing ideas and inspiration to help women reach their goals. In response to short, interactive questionnaires about areas that users find particularly challenging, feedback provides encouragement and tips for overcoming these barriers.

The intervention integrates aspects of acceptance and commitment therapy and cognitive behavioral therapy. These approaches are more often used in face-to-face therapy and have only recently been introduced in eHealth interventions for weight management. The psychology component of the intervention is delivered via video and podcast in 5 modules, with therapeutic techniques and interactive content outlined in detail in a subsequent study. Intervention features and strategies may continue to evolve as the Begin Better intervention uses an adaptive design approach [39] that allows for ongoing improvements to the application following user feedback.

## Discussion

### Principal Findings

In this paper, we systematically report the planning process for a complex, evidence-informed eHealth intervention for preconception health in women above the healthy weight range. We integrated research evidence with BCTs and persuasive technologies that map directly onto behavioral determinants, an approach that is underused and underreported [5]. Incorporating the views of women from a previous study [19] and those of research and professional stakeholders was a valuable addition, allowing for more targeted decision-making about intervention design.

Although traditional weight management programs promote dietary and physical activity changes, the Begin Better intervention also addresses the complex psychological processes that underlie health behaviors. It is widely acknowledged that many of our lifestyle behaviors are governed by automatic responses to contextual cues in our environment—both with food and socially [40]. As humans, we have inherent cognitive limitations in this area [40], often using emotional—rather than logical or rational—decision-making, which can lead to poorer choices. These factors can override individual motivation and intention and have been cited as a reason why many behavior change interventions show only modest effect sizes [41]. We hope that the techniques and engagement strategies used in the Begin Better intervention can address some of these factors.

Our intervention integrates therapeutic approaches that are not often used for health behavior change to assist users in creating the psychological flexibility required to sustain these changes in the long term [42] and within different contexts. Alongside behavior change, the intervention aims to induce cognitive change [43] that may predict the achievement of personal health goals and the maintenance of lifestyle changes.

Some key learning points from various knowledge sources were especially salient in translating theory into an environment for users that would be acceptable, enjoyable to use, and effective. One of the main priorities was the need for simplicity that permeated each step of the planning and development process. This need came through very strongly from interviews and was addressed by keeping behavior changes small, achievable, and flexible for the women to build self-efficacy and motivation for change. Similarly, the interface itself was simplified to 3 key areas that the women would access the most (Nutrition, Mind, and Body). Previous research has also found that users dislike applications that require too much user input [18]. Although greater levels of interactivity predict greater engagement and behavior change [5], these aspects needed to be kept as simple and user-friendly as possible. It was particularly challenging to adapt face-to-face therapy techniques or activities that are often *pen and paper* tasks to a web-based environment. Strategies needed to be chosen and implemented carefully to maintain clarity and ease.

Although not all aspects of step 5—planning, delivery, and implementation—have been explicitly detailed in this paper, an understanding of the factors that influence the wider implementation, scaling, and maintenance of an intervention is crucial for selecting intervention components [44]. The process is highly iterative and context-dependent. In addition, challenges exist in engaging women in preconception planning beyond the current RCT project. At a later stage, undertaking step 6 of the IM protocol—the evaluation plan—will confirm whether the processes undertaken through the development of the intervention have been effective [44].

The outcomes of the Begin Better program are currently being evaluated via an ongoing RCT of 870 women. To be eligible, women will be above the healthy weight range (BMI ≥25 kg/m^2^), intending to become pregnant, and willing to postpone conception for the 6 months of the trial. Women in the intervention group consult with a midwife and dietitian at trial entry, gain access to the full intervention within the Begin Better application, and receive fortnightly individual health coaching sessions. Women in the control group consult with a midwife at trial entry and receive standard preconception care advice within the Begin Better application. At the time of writing, recruitment has commenced with approximately 100 women randomized. Women will complete questionnaires assessing food intake, physical activity, emotional health, and well-being (Depression, Anxiety, and Stress Scale–21) [45] and health-related quality of life (Short Form–12) [46] along with a measure of readiness to change at trial entry and upon completion of the 6-month program. Future research is required to test acceptability and engagement and the relationships between module completion, weight goals, and psychosocial outcomes. Plans are underway to examine the impact of the emotional and affective components of the program, including the mechanistic processes of cognitive change, using scales of stages of change, cognitive flexibility, cognitive fusion, and committed action.

### Strengths and Limitations

Many behavior change interventions are designed with a *one-size-fits-all* approach that fails to account for individual differences [41]. Although the Begin Better application allows for individual values, goals, and habit action plans, personalization may be further integrated in future design iterations. Additional personalization may include motivational SMS text messages tailored to the participants’ individual goals, habits, and coaching sessions.

The need for a pragmatic approach guided our planning methods. The IM protocol involves a highly prescribed, time- and resource-intensive process that could not be implemented in its entirety for this project. Its application can be complicated, as noted by other researchers [10], and consideration of the resources available within the time frames of funding opportunities drove the decision to simplify the process and adapt it to our needs. It is also recommended by experts that intervention developers apply individual approaches in a flexible manner to fit their individual problem and context [31].

Although the intervention evaluation is ongoing, the content was reviewed by specialists in obstetrics, clinical and health psychology, nutrition and dietetics, exercise physiology, and computer science. In addition, potential users reviewed other aspects of the intervention, such as look and feel, and commented on the design decisions, providing insights into how participants may perceive the intervention. However, we did not extensively pilot-test the *live* application with target populations because of time and resource constraints, so new feedback was not collected or integrated.

### Conclusions

The use of an adapted IM process and person-based methods provided us with a clear and tailored approach to developing the Begin Better intervention. In this study, we address a gap in the literature about detailing underlying program mechanisms and outline a process that is potentially transferable to developing other interventions.

## Appendix

Multimedia Appendix 1 The Behavior Change Technique Taxonomy (v1) of 93 hierarchically clustered techniques.

Multimedia Appendix 2 Persuasive system design principles.

Multimedia Appendix 3 Begin Better behavior change objectives, techniques, and practical strategies.

## Abbreviations

BCT: behavior change technique
IM: intervention mapping
PSD: persuasive system design
RCT: randomized controlled trial
