# Supplementary material for: Promoting Health Behavior Change in the Preconception Period: Combined Approach to Intervention Planning
Source: JMIR Form Res. 2022 Apr 28;6(4):e35108. doi: 10.2196/35108 (PMC9100372; doi:10.2196/35108)
Supplement: Multimedia Appendix 1 [file formative_v6i4e35108_app1.docx]

## Behaviour Change Technique (BCT) Taxonomy (v1)

Source [37]: Michie S, Richardson M, Johnston M, Abraham C, Francis J, Hardeman W, et al. The behavior change technique taxonomy (v1) of 93 hierarchically clustered techniques: building an international consensus for the reporting of behavior change interventions. Ann Behav Med. 2013 Aug;46(1):81-95.
PMID: 23512568. doi: 10.1007/s12160-013-9486-6.

| 1. Goals and planning  1.1. Goal setting (behaviour)  1.2. Problem solving  1.3. Goal setting (outcome)  1.4. Action planning  1.5. Review behaviour goal(s)  1.6. Discrepancy between current behaviour and goal  1.7. Review outcome goal(s)  1.8. Behavioural contract  1.9. Commitment  2. Feedback and monitoring  2.1. Monitoring of behaviour by others without feedback  2.2. Feedback on behaviour  2.3. Self-monitoring of behaviour  2.4. Self-monitoring of outcome(s) of behaviour  2.5. Monitoring of outcome(s) of behaviour without feedback  2.6. Biofeedback  2.7. Feedback on outcome(s) of behaviour  3. Social support  3.1. Social support (unspecified)  3.2. Social support (practical)  3.3. Social support (emotional)  4. Shaping knowledge  4.1. Instruction on how to perform the behaviour  4.2. Information about Antecedents  4.3. Re-attribution  4.4. Behavioural experiments  5. Natural consequences  5.1. Information about health consequences  5.2. Salience of consequences  5.3. Information about social and environmental consequences  5.4. Monitoring of emotional consequences  5.5. Anticipated regret  5.6. Information about emotional consequences | 6. Comparison of behaviour  6.1. Demonstration of the behaviour  6.2. Social comparison  6.3. Information about others’ approval  7. Associations  7.1. Prompts/cues  7.2. Cue signalling reward  7.3. Reduce prompts/cues  7.4. Remove access to the reward  7.5. Remove aversive stimulus  7.6. Satiation  7.7. Exposure  7.8. Associative learning  8. Repetition and substitution  8.1. Behavioural practice/rehearsal  8.2. Behaviour substitution  8.3. Habit formation  8.4. Habit reversal  8.5. Overcorrection  8.6. Generalisation of target behaviour  8.7. Graded tasks  9. Comparison of outcomes  9.1. Credible source  9.2. Pros and cons  9.3. Comparative imagining of future outcomes  10. Reward and threat  10.1. Material incentive (behaviour)  10.2. Material reward (behaviour)  10.3. Non-specific reward  10.4. Social reward  10.5. Social incentive  10.6. Non-specific incentive  10.7. Self-incentive  10.8. Incentive (outcome)  10.9. Self-reward  10.10. Reward (outcome)  10.11. Future punishment | 11. Regulation  11.1. Pharmacological support  11.2. Reduce negative emotions  11.3. Conserving mental resources  11.4. Paradoxical instructions  12. Antecedents  12.1. Restructuring the physical environment  12.2. Restructuring the social environment  12.3. Avoidance/reducing exposure to cues for the behaviour  12.4. Distraction  12.5. Adding objects to the environment  12.6. Body changes  13. Identity  13.1. Identification of self as role model  13.2. Framing/reframing  13.3. Incompatible beliefs  13.4. Valued self-identify  13.5. Identity associated with changed behaviour  14. Scheduled consequences  14.1. Behaviour cost  14.2. Punishment  14.3. Remove reward  14.4. Reward approximation  14.5. Rewarding completion  14.6. Situation-specific reward  14.7. Reward incompatible behaviour  14.8. Reward alternative behaviour  14.9. Reduce reward frequency  14.10Remove punishment  15. Self-belief  15.1. Verbal persuasion about capability  15.2. Mental rehearsal of successful performance  15.3. Focus on past success  15.4. Self-talk  16. Covert learning  16.1. Imaginary punishment  16.2. Imaginary reward  16.3. Vicarious consequences |
| --- | --- | --- |
