# Supplementary material for: Promoting Health Behavior Change in the Preconception Period: Combined Approach to Intervention Planning
Source: JMIR Form Res. 2022 Apr 28;6(4):e35108. doi: 10.2196/35108 (PMC9100372; doi:10.2196/35108)
Supplement: Multimedia Appendix 2 [file formative_v6i4e35108_app2.docx]

## Persuasive Systems Design Principles (PSD)

Source [38]: Oinas-Kukkonen H, Harjumaa M. Persuasive Systems Design: Key Issues, Process Model, and System Features. Communications of the Association for Information Systems. 2009;24. doi: 10.17705/1cais.02428.

| Primary Task Support **Reduction**  A system that reduces complex behaviour into simple tasks helps users perform the target behaviour, and it may increase the benefit/cost ratio of a behaviour.  **Tunnelling**  Using the system to guide users through a process or experience provides opportunities to persuade along the way.  **Tailoring**  Information provided by the system will be more persuasive if it is tailored to the potential needs, interests, personality, usage context, or other factors relevant to a user group.  **Personalisation**  A system that offers personalized content or services has a greater capability for persuasion.  **Self-monitoring**  A system that keeps track of one’s own performance or status supports the user in achieving goals.  **Simulation**  Systems that provide simulations can persuade by enabling users to observe immediately the link between cause and effect.  **Rehearsal**  A system providing means with which to rehearse a behaviour can enable people to change their attitudes or behaviour in the real world. Dialogue Support **Praise**  By offering praise, a system can make users more open to persuasion.  **Rewards** Systems that reward target behaviours may have great persuasive powers. | **Reminders** If a system reminds users of their target behaviour, the users will more likely achieve their goals.  **Suggestion** Systems offering fitting suggestions will have greater persuasive powers.  **Similarity** People are more readily persuaded through systems that remind them of themselves in some meaningful way.  **Liking** A system that is visually attractive for its users is likely to be more persuasive.  **Social role** If a system adopts a social role, users will more likely use it for persuasive purposes. Social Support **Social learning**  A person will be more motivated to perform a target behaviour if (s)he can use a system to observe others performing the behaviour.  **Social comparison**  System users will have a greater motivation to perform the target behaviour if they can compare their performance with the performance of others.  **Normative influence**  A system can leverage normative influence or peer pressure to increase the likelihood that a person will adopt a target behaviour.  **Social facilitation**  System users are more likely to perform target behaviour if they discern via the system that others are performing the behaviour along with them. | **Cooperation**  A system can motivate users to adopt a target attitude or behaviour by leveraging human beings’ natural drive to cooperate.  **Competition**  A system can motivate users to adopt a target attitude or behaviour by leveraging human beings’ natural drive to compete.  **Recognition**  By offering public recognition for an individual or group, a system can increase the likelihood that a person/group will adopt a target behaviour. System Credibility Support **‘Trustworthiness** A system that is viewed as trustworthy will have increased powers of persuasion.  **Expertise** A system that is viewed as incorporating expertise will have increased powers of persuasion.  **Surface credibility** People make initial assessments of the system credibility based on a firsthand inspection.  **Real-world feel** A system that highlights people or organization behind its content or services will have more credibility.  **Authority** A system that leverages roles of authority will have enhanced powers of persuasion.  **Third-party endorsements** Third-party endorsements, especially from well-known and respected sources, boost perceptions on system credibility.  **Verifiability** Credibility perceptions will be enhanced if a system makes it easy to verify the accuracy of site content via outside sources. |
| --- | --- | --- |
