# Supplementary material for: Promoting Health Behavior Change in the Preconception Period: Combined Approach to Intervention Planning
Source: JMIR Form Res. 2022 Apr 28;6(4):e35108. doi: 10.2196/35108 (PMC9100372; doi:10.2196/35108)
Supplement: Multimedia Appendix 3 [file formative_v6i4e35108_app3.docx]

**Appendix 3**

Table 1. Begin Better behavior change objectives, techniques, and practical strategies.

| Determinant and subcategories | | Change objectives | Behavior change techniques^a^ | Persuasive system design principles^b^ | Practical strategies to address change objectives |
| --- | --- | --- | --- | --- | --- |
| Information | | | | | |
|  | Poor knowledge of health risks affects action | - Increase knowledge of preconception health | - Information about health, social, environmental, and emotional consequences - Credible source | - Tunneling - Suggestion | - Information about health risks for mother and baby - Highlight benefits of healthy lifestyle before pregnancy - Focus on psychosocial benefits: energy and mood |
|  | Making healthy food decisions to aid weight loss | - Increase nutrition knowledge - Address confusion and food biases - Build healthy food heuristics | - Goal setting - Self-monitoring of behavior - Conserving mental resources - Restructuring the physical and social environment | - Tunneling - Suggestion - Similarity - Liking | - Nutrition information: how to read ingredient lists and nutritional panels, healthy substitutions, and portion control - Target unconscious processes and satiety cues - Reduce cognitive burden of food decisions - Participants: self-monitor food intake - Participants: restructure environment to make healthier decisions easier |
|  | Managing the dilemma of health versus convenience | - Provide fast, healthy alternatives to convenience foods - Build knowledge of physical activity and exercise options | - Problem solving - Self-monitoring of behavior - Behavior substitution - Restructuring the physical and social environment - Avoidance or reducing exposure to cues | - Tunneling - Suggestion - Similarity - Liking | - Simple meal ideas as a counter to convenience foods - Offer easy swaps for unhealthy foods - Information about environmental restructuring to make healthier choices easier to make - Prompt substitution of sedentary behaviors with active ones within similar context - Participants: self-monitor nutrition, use substitutions |
| Personal motivation | | | | | |
|  | Better physical and mental health for my family | - Support all possible health and esthetic motivators | - Goal setting - Action planning - Review behavior goals - Feedback on behavior or outcome - Prompts and cues - Behavior substitution - Identification of self as role model - Framing and reframing - Valued self-identity | - Tunneling - Personalization - Praise - Suggestion - Similarity | - Encourage explicit planning of health behaviors: context, time, and frequency - Praise effort and progress toward goals - Anchor new behavior in current behavior (nudge theory and habit formation) - Harness strength of role modeling healthy behaviors to others - Reframe as positive gains rather than restriction - Participants: self-identify personal values that may motivate, set weight and psychosocial goals |
|  | Taking responsibility for my choices | - Set personal goals (weight and psychosocial) - Monitor food intake and physical activity (self-regulation) | - Discrepancy between current behavior and goal - Review outcome goal(s) - Self-monitoring of behavior - Self-monitoring of outcome(s) of behavior | - Self-monitoring - Simulation - Praise - Reminders - Suggestion - Similarity - Personalization | - Monitor shortfalls in goal attainment, revise goals if necessary - Encourage positive intentions, praise progress - Promote empowerment through influencing own and offspring health - Participants: weigh each week and record, self-monitor via graph of progress, reminders if weight not recorded or module not completed |
|  | Simple, flexible options enhance motivation | - Provide simple solutions that eliminate barriers to behavior change - Improve ability and opportunity to act on motivations | - Action planning - Prompts and cues - Behavior substitution - Habit formation - Graded tasks - Conserving mental resources | - Reduction - Tunneling - Suggestion - Similarity - Liking | - “Nudge” theory: small steps and achievable goals, habit formation - Offer healthier alternatives to convenience foods and flexible activity routines - MI^c^ approach: celebrating partial success, eliciting change talk - Strategies to reduce burden of making food choices, offer flexible options for other family members |
| Social motivation | | | | | |
|  | Feeling the pressure of social expectation | - Encourage principles of “health at every size” | - Information about others’ approval - Social comparison | - Suggestion - Similarity - Normative influence | - Observe principles of “health at every size” - Emphasize benefits beyond weight management - Normative information about others’ behavior |
|  | Interpersonal challenges can affect motivation | - Encourage whole-family health behavior change | - Goal setting (behavior) - Goal setting (outcome) - Social support (unspecified) - Social support (practical) - Social support (emotional) | - Tunneling - Suggestion - Similarity | - Assertiveness training to help manage family members’ expectations - Strategies to influence all family decision makers in making health behavior change - Food alternatives acceptable to family members - Strategies to engage family members in healthy food preparation |
|  | Encouragement and accountability keep motivation high | - Provide ongoing encouragement and motivation | - Review behavior goal(s) - Feedback on outcome(s) of behavior - Social support (practical and emotional) - Social reward - Self-reward - Reward (outcome) - Restructuring the physical environment - Restructuring the social environment | - Tunneling - Personalization - Self-monitoring - Praise - Reminders - Suggestion - Similarity - Social role, learning, comparison, facilitation | - Encouragement and praise via motivational in-application messaging - Emotional support via health coach and opt-in “buddy” system - Activate social support: engage family members and friends to encourage - MI approach that celebrates partial success in achieving goals - Periodic reminders of target healthy behaviors |
| Behavioral skills | | | | | |
|  | Overcoming the mental battle | - Provide psychological support along weight management journey - Increase psychological flexibility | - Problem solving - Social support (emotional) - Information about, and monitoring of, emotional consequences - Reduce unhelpful emotions - Conserving mental resources | - Tunneling - Personalization - Self-monitoring - Rehearsal - Suggestion - Similarity | - Psychological support via ACT^d^, CBT^e^, mindfulness, and stress management strategies - Personal strategies to manage expectations, setbacks, relapse prevention, coping planning, and reframing unhelpful thoughts - Encourage monitoring of emotions after eating healthy food or exercising - Promote positive psychological benefits |
|  | It would be easy with better time management | - Provide alternatives to manage time commitments | - Goal setting - Problem solving - Action planning - Prompts and cues | - Suggestion - Similarity - Social role - Social learning | - Encourage simplicity in making healthy food and exercise part of everyday schedule - Offer alternatives that consider family and work commitments |
|  | Taking care of myself is important as I value my health | - Reinforce importance of self-care | - Information about health, social, environmental, and emotional consequences | - Suggestion - Similarity | - Emphasize health gains beyond weight loss - Stress management and mindfulness skills, including mindful eating - Strategies to prioritize own needs above those of family members |
|  | I need more inspiration than information | - Inspire with easy, healthy, tasty meal ideas | - Instruction on how to perform a behavior - Demonstration of the behavior | - Suggestion - Similarity - Liking - Social learning | - Healthy, colorful, inspiring recipes - Exercise regimes specifically designed for women with overweight or obesity |
|  | Believing in myself to change my lifestyle | - Develop  self-efficacy beliefs | - Goal setting (behavior) - Action planning - Verbal persuasion about capability - Focus on past success - Self-talk | - Tunneling - Personalization - Suggestion - Similarity | - Build self-efficacy about performance of health behaviors, assert success, argue against self-doubt - Build action and coping  self-efficacy - Prompt previous successes in health behaviors - Support “change talk” with motivational messages,  focus on gains |
| Environmental factors | | | | | |
|  | Affordability limits access to healthy options | - Provide options that are easily accessible and cost-effective | - Instruction on how to perform a behavior - Demonstration of the behavior | - Reduction - Suggestion - Similarity - Social learning | - Exercise regimes that can be done in the home without special equipment - Easy recipes using simple, affordable ingredients |

^a^Behavior change techniques are coded in accordance with the 16 hierarchically clustered groups of the Behavior Change Technique Taxonomy (v1) [28].

^b^System credibility support is a set of 7 persuasive system design model principles used throughout: trustworthiness, expertise, surface credibility, real-world feel, authority, third-party endorsements, and verifiability.

^c^MI: motivational interviewing.

^d^ACT: acceptance and commitment therapy.

^e^CBT: cognitive behavioral therapy.
